# Exploration of Effects of Graduated Compression Stocking Structures on Performance Properties Using Principal Component Analysis: A Promising Method for Simultaneous Optimization of Properties

**DOI:** 10.3390/polym14102045

**Published:** 2022-05-17

**Authors:** Hafsa Jamshaid, Rajesh Kumar Mishra, Naseer Ahmad, Muhammad Nadeem, Miroslav Muller, Viktor Kolar

**Affiliations:** 1 Faculty of Textile Engineering, National Textile University, Faisalabad 37610, Pakistan; hafsa@ntu.edu.pk (H.J.); naseerahmad@ntu.edu.pk (N.A.); mnadeem6258@gmail.com (M.N.); 2 Department of Material Science and Manufacturing Technology, Faculty of Engineering, Czech University of Life Sciences Prague, Kamycka 129, 165 00 Prague, Czech Republic; muller@tf.czu.cz (M.M.); vkolar@tf.czu.cz (V.K.)

**Keywords:** graduated compression stockings, medical textile, principal component analysis, thermo-phycological comfort, inlay yarn, insertion density

## Abstract

This paper focuses on the comfort properties of graduated and preventive compression stockings for people who work long hours in standing postures and for athletes for proper blood circulation. The present study was conducted in order to investigate the effects of the yarn insertion density and inlaid stitches on the performance of the compression stockings. The effects of these parameters on the thermo-physiological comfort properties were tested with standard and developed methods of testing. All compression stockings were maintained with class 1 pressure as per German standards. The structural parameters of the knitted fabric structures were investigated. The stretching and recovery properties were also investigated to determine the performance properties. The theoretical pressure was predicated using the Laplace’s law by testing the stockings’ tensile properties. The compression interface pressures of all stockings were also investigated using a medical stocking tester (MST) from Salzmann AG, St. Gallen, Switzerland. Correlation between the theoretical pressures and pressures measured using the MST system were also assessed. The current research used a multi-response optimization technique, i.e., principal component analysis (PCA), to identify the best structure based on the optimalization of the above-mentioned properties. The results also revealed that samples with higher insertion density levels exhibit better comfort properties. The results showed that sample R1 was the best sample, followed by R2 and P. In addition, all developed stocking samples exhibited better comfort properties than the control sample from the market.

## 1. Introduction

The successful usage of compression garments has been in practice for a long time in order to prevent medical issues such as scarring, edema and deep vein thrombosis (DVT) in hospitalized patients, either immediately after surgery or because of immobility due to a medical illness [1,2]. DVT can be prevented with the use of compression stockings instead of drugs. Drugs can cause bleeding, which is a primary concern in patients who have undergone surgery. Compression can be applied using bandages and elastic compression hosieries (ECHs). Compared with bandages, ECHs are generally more in demand due to their convenience and ease of care. Compression hosiery products can be developed using circular and flat knitting machines. These are functional products that combine knitting technologies to meet healthcare requirements. Graduated compression stockings (GCS) help prevent the formation of blood clots in the legs by applying varying amounts of pressure to different parts of the leg [3,4]. Compression stockings are known as an effective nonsurgical choice, exerting gradual pressure to prevent and to treat venous disease in legs [5]. In the field of healthcare, compression stockings play a significant role, being used as a first line of treatment to manage the increasing intensity of varicose veins or chronic venous diseases at the initial stages [4,5,6]. Varicose veins are abnormal, whereby dilated blood vessels develop due to weaknesses in the vessel walls. This appears as swollen, twisted clusters or clots of blue or purple veins, which cause the blood to back up and pool inside the vein and make a new route [7]. Compression stockings exert an external pressure on lower limbs, reduce the vein diameter and increase the venous blood flow velocity, which helps the vein valves to function properly [8]. Researchers concluded that for compression stockings, ≥20 mmHg is an effective range of optimum pressure values that cause the fastest venous flow in various regions. There are different values for the ankle, calf, knee, lower thigh and upper thigh regions, which are 18 mmHg, 14 mmHg, 10 mmHg and 8 mmHg, respectively [9].

Compression stockings can be worn for an entire day because they maintain compressive pressure and do not lose their elastic stretch recovery over many hours. Changes in body posture significantly influence the skin pressure profile [10]. Nowadays, the biggest challenge for compression products and new devices is to make the compression more comfortable and acceptable for patients [11]. Usually, comfort is related to three aspects, namely thermo-physiological, sensorial and psychological factors. The sweat absorption and drying abilities are the main components of thermo-physiological comfort. It has been observed that the structural parameters of the knitted fabrics play an important role in determining the moisture management and thermal properties [12,13]. Researchers investigated the effects of the materials and structural parameters of compression stockings on the skin pressure distribution via material testing and wear trials [14]. Bruniaux et al. developed different models to study the effect of yarn on compression [15].

Cieślak et al. analyzed the impacts of pressure measurement methods along with two types of knitting structures, i.e., warp knitting and weft knitting, on the compression values using the same materials and concluded that that pressure values were lower in the warp-knitted fabric as compared to the single-jersey weft-knitted fabric in both vivo and vitro tests [16]. Troynikov et al. studied the effects of the physical attributes of warp-knitted tricot and single-jersey weft-knitted products on compression values. They concluded that the degree of compression pressure directly influences the construction and fit of the garment, the structure and physical properties of the material and the size and shape of the garment [17]. Liu et al. studied plain and rib structures developed on circular sock machines and conducted in vitro and in vivo testing of the compression pressure, finding consistent results [18]. Partsch et al. studied the compression and stiffness parameters of different compression stockings that involved European standards classes 1, 2 and 3 with pressure ranges of 15–21, 23–32, 34–46 mmHg, respectively [19]. Alisauskiene et al. investigated the effects of the linear density and insertion density of double-covered inlay yarn on the mechanical behavior (such as the washing and drying shrinkage percentage and compression behavior) of weft-knitted orthopedic supports [20]. Sarı et al. investigated the effects of process parameters, i.e., elastane yarn fineness, feeding tension and tightness factor, on the pressure of compression stockings [21]. Similarly, in another study researchers investigated the effects of the elastane yarn count on the extensibility and bursting strength of stockings [22]. 

The development of compression stockings is a complex process, and most of the scientific knowledge available is primarily focused on the study of readymade stockings, pressure evaluations and material properties [4,14,19,23,24]. Although compression stockings have gained popularity in recent years, the knowledge available on their performance in terms of thermo-physiological comfort remains scarce. 

The aim of the present study was to develop compression stockings with different patterns and structures by changing the inlay yarn density and laid-in stitches while keeping the compression class constant. Compression testing was carried out using two different methods and their correlation was established. Stretch and recovery% values were also investigated for all samples. Additionally, the effects of the inlay yarn density and laid-in stitches on thermo-physiological comfort properties were also studied. All of the developed samples were compared with the control sample from the market. Furthermore, the current study focused on the optimization of the above fabric properties using principal component analysis (PCA). The study is expected to provide solutions for more comfortable compression stockings for patients.

## 2. Materials and Methods

### 2.1. Materials

Compression stocking samples were purchased from different commercial brands, e.g., TED^®^ (D) and Sockey^®^ (C) of Germany and China, respectively. The purchased samples were of compression class I, which is more commonly referred to as the standard in clinical application [25]. Class I is used for minor varicose veins, venous insufficiency and mild edema. All of the samples available on the market have a plain–single-jersey (1 × 1) structure (without insertion). Such a sample was used as a control for comparison purposes. The yarn specifications of the developed samples are shown in Table 1. The analysis was performed with great precision and accuracy under standard atmospheric conditions.

**Figure 1:**
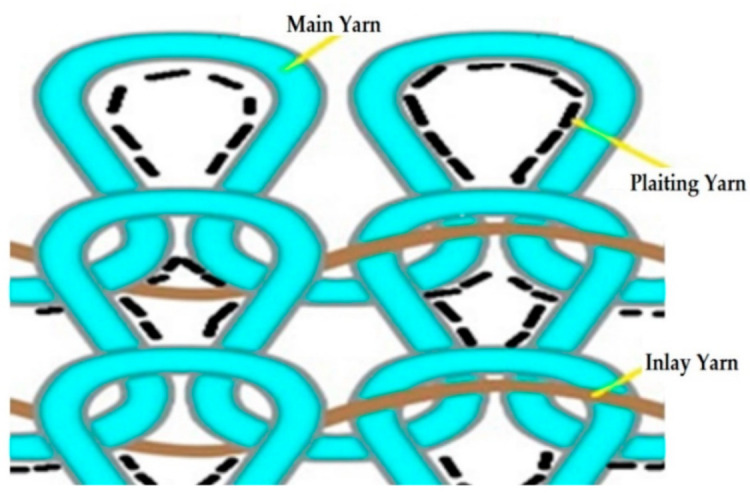
Schematic diagram of yarn.

Due to the different machine settings, V-shaped socks were produced using three types of yarn, i.e., main, plaiting and inlay yarns. In this research work, three types of fiber materials were used, i.e., 100% polyamide (PA 6.6) filament yarn as the main yarn, polyurethane (PU(Lycra^®^))/PA6.6 single-covered (SCV) yarn as a plaiting yarn or ground yarn and PU(Lycra^®^)/PA6.6 double-covered (DCV) yarn as an inlay yarn, as mentioned in Figure 1. All yarns were imported from China.

The yarn specifications are given in Table 1.

### 2.2. Methods

**Figure 2:**
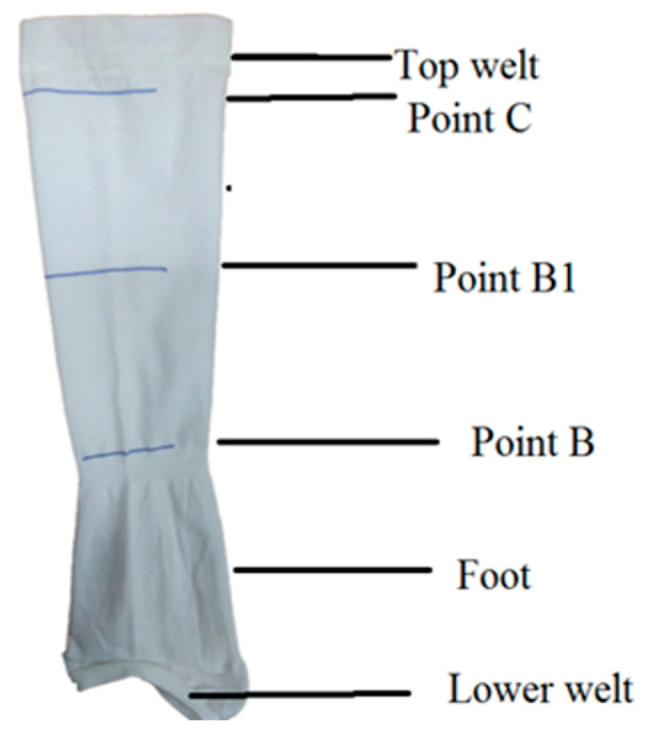
Graduated compression stocking with different compression levels.

Compression stockings were manufactured using a professional circular compression knitting machine. The machine was a Merz CC4-2 instrument from Germany, with a 12 cm diameter, E 24 machine gauge, 360 needles and 4 feeders, including two furnishers. The first feeder has 10 fingers, while the second, third and fourth feeders have 8 fingers. For the development of compression stockings, inlay yarn was fed at the first and third feeders, while the plaiting yarn and main yarn were fed through the second and fourth feeders, respectively. At feeder no. 1, in finger 2 and feeder 8, nylon filaments yarn was used for foot formation. At feeder no. 3, double-covered yarn in finger no. 4 and nylon filament yarn in finger 6 were fed for top welt formation. The inlay yarn arrangement is the main decisive factor in the manufacturing of different knitted structures. Compression stockings were made in plain and mock rib structures. A total of six samples were developed, representing the plain, rib 2 × 2 and rib 1 × 3 structures. The samples differed according to their inlay yarn insertion pattern (laid in stitch) and structure. In the first 3 structures, with sample codes P, R1 and R2, the inlay yarn was inserted after one empty course, while in other three samples with codes IP1, IR1 and IR2, the inlay yarn was inserted in each course. At the start the welt was formed and after 2–3 steps or courses the welt was completed. The graduated pressure regions denoted as points C, B1 and B in Figure 2 were manufactured as per the program. In the final step, the foot portion was manufactured.

The compression stockings were made in plain and mock rib structures (longer inlay threads floating at the back, forming a mock rib visual effect). The samples and their codes are listed in Table 2.

Loop configurations and structures and fabric simulations were constructed using the SDS-ONE APEX platform (Shima Seiki, Wakayama, Japan), as shown in Table 3 [26]. The corresponding microscopic images from the front and back views were also taken using an OPTIKA C-B 10 microscope (via Rigla, 30 24010 Ponteranica BG-Italy, SN 536495) with an M-144 fixed microscope adaptor. 

All of the developed samples were of compression class 1 (C1), i.e., 18–21 mmHg and medium (M) size. The graduated pressure percentages for the class 1 samples were in line with Quality Assurance RALGZ 387/1 [27], as shown in Table 4. 

**Figure 3:**
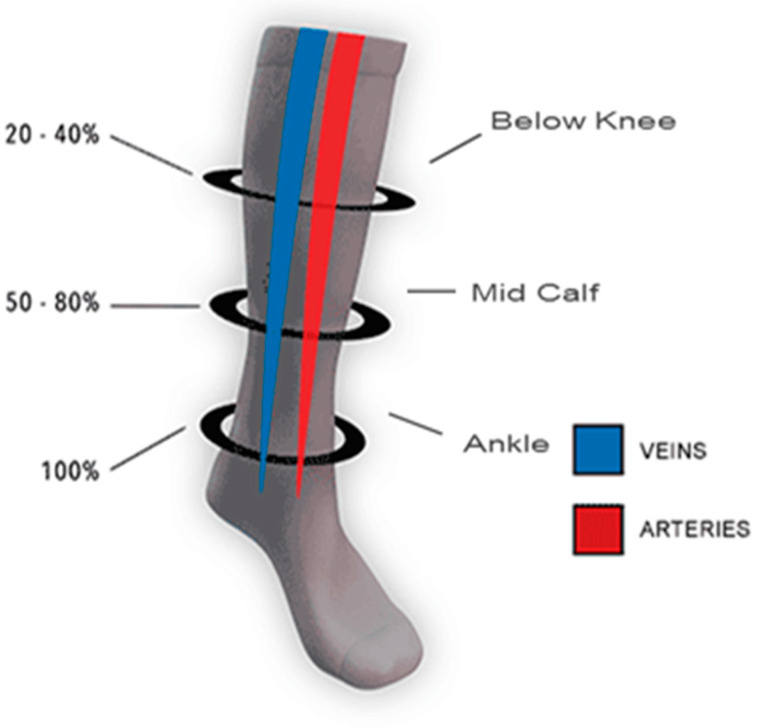
Pressure levels in the compression stocking.

In the present study, the developed calf (below-knee) stockings contained 3 pressure regions, i.e., B, B1 and C. The pressure levels are shown in Figure 3. 

#### Washing Method

The samples were washed according to Australian guidelines AS-2001:5-2005. Mild detergent was used in water and a soapy solution was prepared. The washing agent should not contain any bleaching agent, additives or chlorine because such substances weaken the elastic behavior of compression socks [28]. The samples were soaked in mild detergent solution for 5 min. Next, the stockings were shaken in the liquid for 2–2.5 min and then rinsed off with water. The excessive water was removed from the stockings by pressing them between the palms gently and then they were dried for 24 h on a flat surface. The washing and drying process was performed at room temperature. All testing was performed after washing. 

### 2.3. Testing

#### 2.3.1. Yarn Linear Density 

Linear density values of the yarns were tested as per ASTM D 1059-17, and the numbers of filaments were counted using a microscope (Beck London 35288, Model 47, London, UK).

#### 2.3.2. Physical Properties

Testing was performed after washing and at all reference points, as shown in Figure 2. These explanations are given only for point B, as this is the main reference point of interface pressure and also shows the maximal extension during movement. An analysis was performed of the physical properties of the stockings, i.e., courses per centimeter (CPC), wales per centimeter (WPC), areal density (GSM) and thickness. Courses and wales per cm were counted using counting glass as per laboratory practice. Thickness values were measured as per ASTM D1777, using a 99-0697 thickness tester (Framincham, MA, USA). To determine the areal density values (g/m^2^) of stockings, a model JH-10-36 GSM cutter (Jenhaur Co. Ltd., Taipei, Taiwan) was used to cut samples according to ASTM D 3776. 

#### 2.3.3. Stretch and Recovery Percentages

The stretch and recovery ability is very important for properly fitting socks. Stretching is also directly linked to the comfort of a garment, as it relates to the ease of wearing a garment. The optimum level of stretchiness is a basic requirement in knitted stockings to support blood flow in the legs and feet. This is one of the most important performance characteristics. Poor stretching results in a poor fit, leading to performance shortcomings such as discomfort. Compression stockings should be designed such that they maintain a uniform interface pressure gradient over the limb for effective recovery results [29,30]. Stretch measurements of all compression stockings were performed on a CETME Attrezzature Per Calzific instrument from Reggio Em., Italy, as per ASTM D 2594. The stretchiness is the amount of extension of the fabric under a determined force, while the elasticity is the fabric’s recovery ability after stretching, which determines the dimensional stability of an elastic garment.

Stretch and recovery % values were calculated using Equations (1) and (2).

The stretch percentages were calculated using Equation (1):

(1) Fabricstretch%=(B−A)/A×100

where *A* is the original distance between marked points prior to the application of tension, *B* is the distance between benchmark points on the specimen under tension. 

For the calculation of the recovery percentages, Equation (2) was used:

(2) Fabricrecovery%=(B−D)/(B−A)×100

where *A* is the original distance between marked points prior to the application of tension, *B* is the distance between benchmarks on the specimen under tension and *D* is the distance between benchmarks after the release of tension. 

#### 2.3.4. Shrinkage %

All the samples were tested in circumference and transversal directions after washing.

#### 2.3.5. Compression Testing

**Figure 4:**
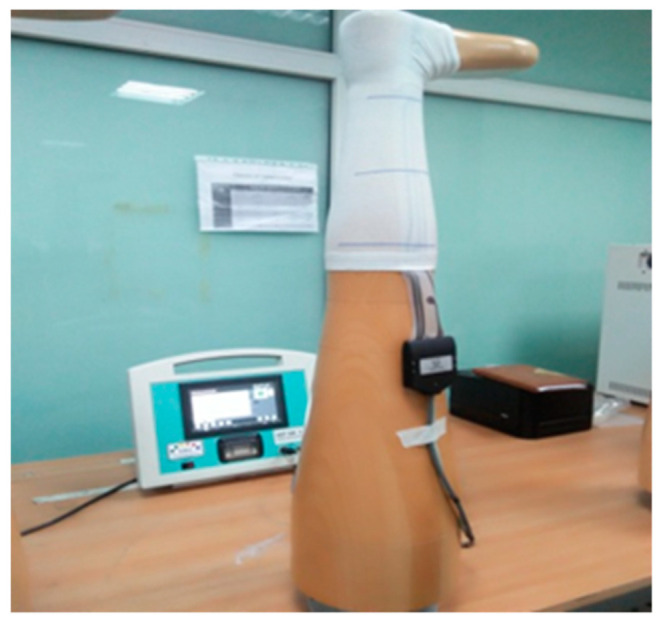
A medical stocking tester (MST) and MK V pressure measurement device.

Compression testing can be performed in two ways in vivo (direct or wearer method) and in vitro (indirect or model method) using different instruments, such as a HOSY system, tensile testing device, HATRA and medical stocking tester (MST). In the present research, an in vitro method was adopted to evaluate the compression pressure values. A Sazlmann AG stocking measuring device available in different MST models (e.g., MST, MST II, MST III, MST IV and MST V models) (Salzmann AG, St Gallen, Switzerland, MST MK V), as shown in Figure 4, was used in the present research. The measurement principle of MST is based on the use of a pressure transducer. The compression testing was performed according to 387/1-2008, using quality and testing specifications for medical compression hosiery prescribed by RAL German Institute for Quality Assurance and Labeling [27]. A pneumatic probe or sensor was placed between a wooden leg and stocking samples. Compression pressure values were measured at the pressure points, e.g., the ankle (B), gaiter (B1) and calf (C). The samples were placed vertically along a wooden leg model with a circular cross-section. In compression garments, mmHg is commonly used as the unit of compression pressure to define the pressure dosage (1 mmHg = 133.322 Pascal). All pressure values were noted in mmHg. The pressure values were measured when each reading was stable. Three readings were taken at each point and the average was used for analysis. Three measurements were taken at each measurement point after an interval of three minutes. 

All compression stockings were designed on the principle of elastic compression. Compression stockings on the leg generate the pressure with which the stocking presses the leg. Laplace’s law explains how the pressure is exerted by stockings on the limb. It has been reported that pressure exerted by a strip on the surface of a human leg can be determined by Laplace’s law. This law is now widely used to explain and assess the pressure delivered to a limb of a known radius by a fabric under known tension [31,32]. Equation (3) is shown below:

(3) P=T/r

where *P* denotes pressure (Pa), *T* is the tension of the compression material on the leg (Nm^−1^), *r* is the radius (m) of the limb surface on which it is applied and *W* is the width in meters. Mathematical models were developed on the basis of Laplace’s law. Compression measurements were also carried out using a tensile testing device to compare the two methods. A tensile testing machine (LLOYD, LRX Plus, Largo, FL, USA) was used for measurements of fabric tension during extension [21]. Extension in the course direction was chosen because compression stockings are stretched in the transversal direction to generate pressure during wear. The tensile properties were evaluated at pressure point “B” using the same machine settings. The device gauge length was set to 86 mm, while extension using a constant traverse speed of 100 mm/min and load cell of 100 N was used. The specimen width was 8 cm and the radius was 4 ± 0.4 cm for all samples. A comparison was made between calculated pressure and experimental measurements using an MST instrument. 

#### 2.3.6. Thermo-Physiological Comfort Properties

The air permeability (AP) of all samples was tested using an air permeability tester (M021A, SDL Atlas, Newark, NJ, USA) as per the ISO-9237 standard under a pressure of 100 Pa. In total, ten readings were taken and the average was calculated.

Moisture management of the samples was checked using a moisture management tester (M 290 SDL Atlas, Newark, NJ, USA) as per the AATCC-195 standard. In total, three readings were taken and their average was calculated.

Thermal properties of samples were checked by Permetest, Liberec, Czech Republic as per standard ISO-11092. Five readings were taken, and average was calculated.

All experiments were carried out at a standard atmosphere, i.e., 21 ± 1 °C temperature and 65 ± 2% relative humidity in compliance with ISO 139:2005 standard.

#### 2.3.7. Data Analysis

Means and standard deviations were computed for all test results. In the process of manufacturing a standardized product, variability is considered the main hurdle in achieving the desired product quality. Different researchers have proven the process optimization technique as the best solution for not only improving the quality but also reducing error in the development of the final product. The optimization techniques include grey relational analysis (GRA), principal component analysis (PCA), desirability function, artificial neural network (ANN), fuzzy logic, data development and analysis and weighted signal-to-noise (WSN) approaches [33,34]. PCA explains the variation in a set of parameters through a few linear combinations called principal components in order to reduce the original data set and refine the linear relations that exists among the variables. Each principal component explains the percentage of overall variation among the variables. These principal components are ranked in descending order, from the maximum to the minimum. In PCA, multiple response variables are converted using eigenvalues as weights into a single response, called a multi-response performance index (MRPI), which is computed by adopting the following procedure. MRPI values are computed by first computing the signal-to-noise and normalized signal-to-noise ratios of each response using the standard method described in earlier studies [35].

The samples and their codes are shown in Table 2. The finishing experiments were conducted in three independent replicates for each sample and the data reported are the mean values of three readings. In the present study, PCA was employed to identify the best parameters for samples subject to the simultaneous optimization of multiple responses in the development of stockings, including compression by MST, air permeability, thermal resistance, overall moisture management capacity (OMMC), stretch %, recovery %, shrinkage %, thickness and areal density.

## 3. Results and Discussion

The physical features, dimensional properties (i.e., shrinkage, stretch and recovery %) and thermo-physiological comfort properties of the control sample from the market (denoted as C) and of the developed samples (P, R1, R2, IP1, IR1, IR2) were analyzed.

In total, 6 sets of experimental runs and corresponding experiments on the nine responses were performed, including compression by MST (mmHg), air permeability (AP in mm/sec), thermal resistance (TR in m^2^K/W), OMMC, stretch (%), recovery (%), shrinkage (%), thickness (mm) and areal density (g/m^2^), with the results given in Table 5.

**Figure 5:**
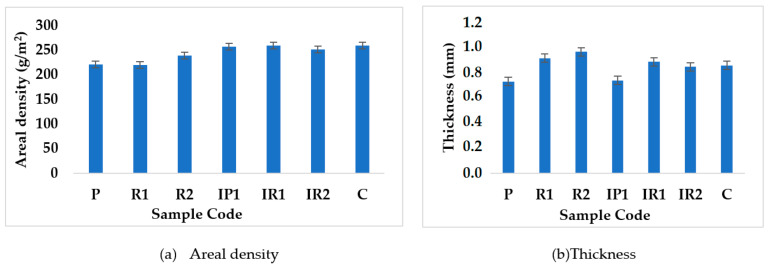
Physical properties of graduated stocking at point B.

The fabric areal density and thickness values of all samples (P, R1, R2, IP1, IR1, IR2 and C) are compared in Figure 5.

The areal density and thickness of the fabric are important properties. The areal density and thickness were measured and compared on the basis of the pattern of insertion of the inlay yarn and structure, as shown in Figure 5. Samples IP1, IR1 and IR2 were found to have higher areal density or GSM values due to the insertion of inlay yarn in every course, which led to increased fabric weight per unit surface area. As the number of tuck stitches increases, the accumulation of yarn in the tucking zones increases, which leads to increases in weight. In 1 × 1 samples, the inlay yarn was tucked in one wale and floated in the next wale consecutively. In 2 × 2 samples, the inlay yarn was tucked into two wales followed by two floating loops. In 1 × 3 samples, the inlay yarn was tucked in one wale followed by three floating loops. The effect of the thickness on the pattern was not significant. Patterns with lower insertion density values were found to have higher levels of thickness. This may be attributed to the increased bulkiness of the samples. By increasing the number of missing stitches in the inlay yarn, the thickness was increased, as was observed by other researchers [36]. The properties of the developed samples were compared with the control sample (C) from the market.

### 3.1. Stretch % and Recovery %

**Figure 6:**
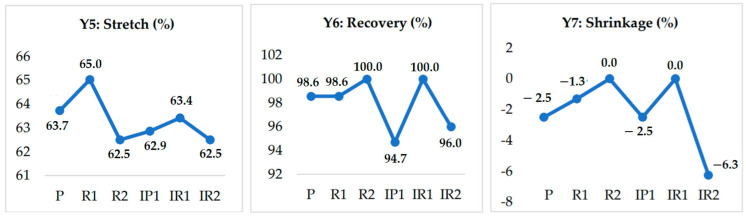
Dimensional properties of compression stockings.

As the inlay yarn insertion density increased, the stretch % and recovery % decreased. This may have been due to the tight structures, whereby movement of yarn was restricted. This trend is illustrated in Figure 6. The stretching is inversely proportional to the tightness. As the structure becomes tighter, the level of stretching decreases. In the 1 × 1 structure, 1 floating loop length alternatively appeared in the wale, while in the other wales it was tucked. A tucked wale is less stretchable than a floating wale. The reason for the lower level of stretching was the alternative tuck pattern of the inlay yarn in the wale direction. In the 2 × 2 structure, the two consecutive floating loops of inlay yarns led to greater stretching in the fabric. These two floats do not pass through any loops when the force is applied. Thus, the fabric stretches without any restriction from the knitted loops. In the 1 × 3 structure, there were three consecutive floating loops of inlay yarn, which were then tucked in the same wale. This arrangement increases the restriction to movement and reduces the extensibility [36]. 

### 3.2. Shrinkage %

Shrinkage is also an important factor influencing the dimensional stability during washing. Due to the elastomeric yarns, the fabric dimensions changed. The circumferences of the stockings were reduced after washing due to shrinkage in the knitted structures. The shrinkage is reduced if a structure is knitted with a smaller number of inlay yarns. It can be observed that with an increase in inlay yarn, the circumference of the stocking is reduce. Shrinkage of less than +6% is not considered significant. Normally, increasing the tuck loop results in higher stability and shape retention in knitted structures [37]. The recovery % values of all tested samples were found to be above +94%, which implies that the compression textiles sustained their properties after washing.

### 3.3. Compression Measurement

The compression pressure measurements were performed using two methods. All samples satisfied the requirements for class 1 compression, irrespective of the inlay yarn insertion density and structure.

Using the MST method, compression testing was performed on each pressure point in the control and developed samples. Using the tensile testing method, compression was measured only at point B in the developed samples. Point B is the most crucial pressure point for compression stockings. As we move from point B to B1 and B1 to C, the structure becomes wider as the stitch length increases; thus, the compression value decreases. This phenomenon is known as gradual compression. During knitting and testing, it was verified that the compression of all the samples remained gradual and within the class 1 pressure range according to the RAL 387/1 standard. This pressure range helps the blood to flow in the upward direction due to the lower pressure at higher points.

From the results, it is clear that a knitted pattern where inlay yarn is inserted in every course has better compression properties. Samples where the inlay yarn is inserted in alternating courses generate lower levels of compression, as supported by other researchers [36,37]. Xiong et al. [38] also stated that the inlay yarn provides the required level of compression in elastic fabrics, while the main yarn provides the stiffness and thickness. To achieve a higher level of compression, it is necessary to increase the density of the inlay yarn. Table 6 and Table 7 summarize the results. 

The pressure was calculated using Laplace’s equation and was measured using MST (R = 4.07 cm) compression at point B [39,40].

**Figure 7:**
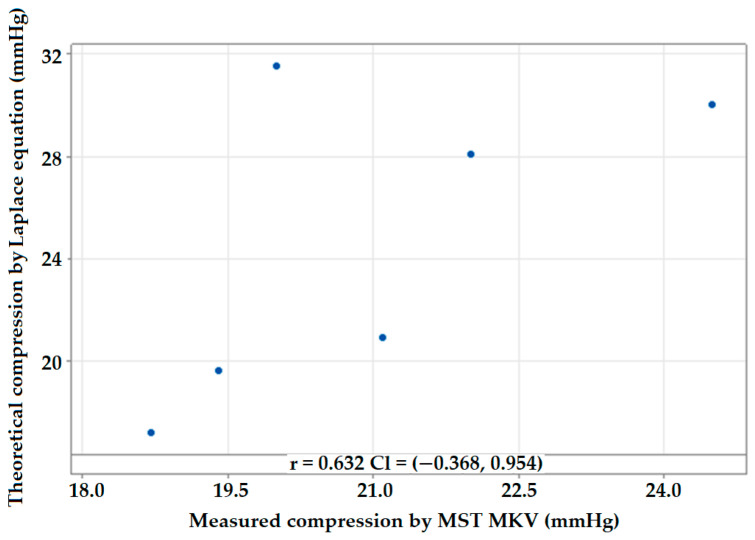
Correlation between measured and calculated compression pressure.

The deviation percent was calculated as the difference between the measured compression using the MST and pressure calculated using Laplace’s equation. The results in Table 6 show that the deviation values when applying Laplace’s equation for point B ranged from ±0.7% to ±57.6%. There was significant deviation when applying Laplace’s equation in all samples when compared with the practically measured compression values using the MST method. The deviation was higher in points where the inlay yarn insertion was increased. This is because more pressure is exerted with a higher inlay yarn insertion density. The theoretical pressure values calculated using the equation did not accurately predict the values found using the experimental method due to several limitations, as explained by other researchers [41,42,43,44]. The correlation coefficient of the interface pressure using both experimental and theoretical methods was found to be 0.632, as shown in Figure 7. Lower R values depict smaller correlations between the two methods.

### 3.4. Comfort Properties

#### 3.4.1. Air Permeability

The air permeability is an important property with respect to comfort, especially regarding the breathability of compression stockings. These stockings are worn for the majority of the day, meaning a lack of comfort negatively affects performance. Under real conditions during wear, stretching of the stocking would cause increased interloop spaces, which would also affect air permeability. From point B to B1 and from B1 to C, the structure becomes wider the and stitch length increases. As a result, the air permeability increases.

**Figure 8:**
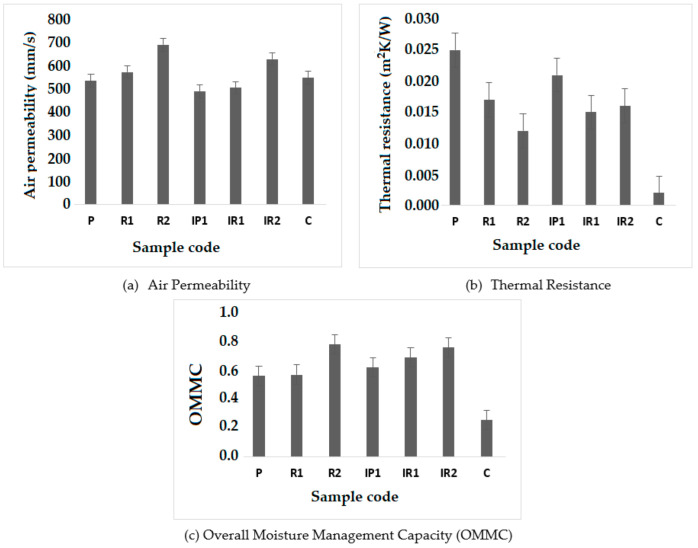
Thermo-phycological comfort properties of the developed stockings.

From the Figure 8a, it is clear that stocking samples without insertion show higher air permeability due to the lack of insertion of the inlay yarn, as compared to samples 4–6, where insertion of the inlay yarn is performed in each course. The inter-yarn pores are the most important components, which influence the porosity of the fabric structure and facilitate the passage of air.

The type of structure also has a strong influence on the air permeability, as it affects the spaces in the loop, which determine the porosity and ultimately the air permeability. This can be seen in the optical or microscopic images of fabrics shown in Table 3.

Due to the arrangement of tucked and missed stitches in the inlay yarn in the developed knitted structures, a visible difference in air permeability can be observed. In the 1 × 1 structure, the inlay yarn is tucked in the wales alternatively with a floating loop. In the 2 × 2 rib structure, the inlay yarn is fed in two wales followed by two floating loops. In the 1 × 3 rib structure, the inlay yarn is fed into one wale followed by three floating loops. Due to the longer float length of the inlay yarn, spaces in the loops and the pore size increase, which leads to higher air permeability. Missed stitches in knitted fabrics result in lower surface area and allow the air to pass through easily. The commercial control sample (C) had a 1 × 1 structure without insertion and had comparable air permeability to the developed samples.

#### 3.4.2. Thermal Resistance

While moving from point B to C, the structure becomes wider as the tightness decreases and stitch length increases. This results in emptier spaces in the structure at higher points, while the thermal resistance decreases.

It can be observed from Figure 8b that samples without the insertion of inlay yarn in every course show higher thermal resistance than samples with insertion. The reason may be due to the lower thickness of theses samples. The fabric thickness has a profound effect on the thermal resistance. The type of structure has a significant effect on the thermal resistance. More compactness in the structure results in higher thermal resistance, as stagnant air is entrapped in small pores. Stagnant air has the lowest thermal conductivity when compared with other fibers and solids. It has been previously reported that dead air pockets in knitted structures increase the thermal resistance of the fabric [45]. 

The thermal resistance of stagnant air is higher than that of fiber. From the microscopic images, it is evident that sample R2 has bigger pores. Therefore, air cannot be entrapped in it due to the longer floating loops as compared to R1 and P. The accumulation of yarn due to tucked stitches affects the dead air pockets, which increases the thermal resistance. As the float length increases, the dead air pockets decrease. The commercial sample had lower thermal resistance than the developed samples.

#### 3.4.3. Overall Moisture Management Capacity (OMMC)

Appropriate moisture management is essential for stockings to prevent the formation of wet conditions, which leads to reduced tolerance of the human tissues to shear stress and friction. A higher moisture content and sweating lead to uncomfortable conditions for the wearer. Moisture management is a very important property facilitating the evaporation of generated sweat. Overall, the developed stockings exhibited reasonably good moisture management properties. The samples showed OMMC values higher than 0.5, which were in the good (0.4–0.6) and very good grade range (0.6–0.8).

The commercial sample had an OMMC value lower than 0.5, making it uncomfortable to wear in summer season. 

From the Figure 8c, it can be observed that the samples with the insertion of inlay yarn in every course exhibit lower OMMC values than samples without insertion. This is due hindrance of the vapor transfer due to the presence of tuck loops. These loops may stop the flow of water in capillary spaces. The 1 × 1 structure shows a lower OMMC value than the other structures, e.g., 2 × 2 and 1 × 3. Due to the alternating tuck and miss stitches, the transmission of vapor decreases, which leads to an increased wetting time and decrease in spreading speed. With more tuck points, the 2 × 2 structure exhibits a higher OMMC value than the 1 × 1 knitted structure. This is due to the higher number of tuck points, which act as capillary channels for moisture transmission from inner to outer surfaces. As the density of the missed stitches increases, the OMMC also improves.

#### 3.4.4. Principal Component Analysis (PCA) of the Results

The multi-response optimization of the nine responses was computed by performing the following steps.

In the first step, the signal-to-noise ratio of each response in each run was commuted depending on the quality characteristics by adopting the standard method. The results are shown in Table 8. For comparison purposes, the normalized signal-to-noise ratios of all responses were computed depending on the quality characteristic, e.g., the higher the better, the lower the better or the more nominal the better. In the present study, the quality characteristics are compression MST (mmHg), AP (mm/s), OMMC, stretching (%) and recovery (%). For these characteristics, the responses were taken as the higher the better. However, for thermal resistance (TR in m^2^K/W), shrinkage (%), thickness (mm) and arial density (g/m^2^), the responses were set as the lower the better. Normalized signal-to-noise ratios are given in Table 9.

As discussed earlier, PCA explains the variation in a set of parameters via linear combinations. Fewer variables are preferred in order to reduce the original linear relations between the variables. Each principal component (PC) corresponds to a percentage of the total variation. In this study, PC1 represents the linear combination that explains the maximum variance (43.6%), followed by PC2, PC3 and PC4 (52.1%). The last five PCs (PC5–PC9) contribute to less than 5% of the variance. The first four PCs represent almost 96% of the total variance among the data set, as shown in Table 10. The next step is to decide the number of PCs retained.

As per the Kaiser–Guttman principle, the first three principal components (PC1, PC2, PC3) with eigenvalues greater than one were kept for further analysis (Table 10).

**Figure 9:**
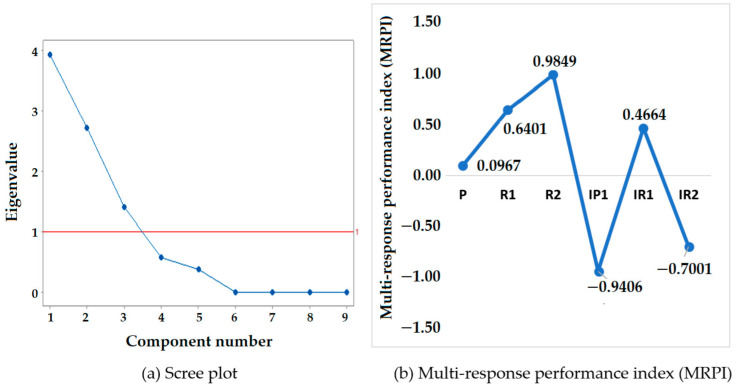
Scree plot and MRPI values.

The normalized signal-to-noise ratios were taken for the analysis using Minitab 17. The first three components had eigenvalues greater than one, as shown in Table 10. The scree plot shown in Figure 9a was taken for further analysis. The normalized S/N ratios were used to perform the multivariate analysis using Minitab 17. The first and second principal components had eigenvalues greater than one. Thus, they were considered for further analysis. The principal component analysis matrix is given in Table 11. The results are presented in Figure 9b. 

From the results given in Table 11, the order of the multi-response performance index (MRPI) values for the second, third and first runs was obtained as first, second and third highest, respectively, as illustrated in Table 12 and Figure 9b. This implies that samples R1, R2 and P may be more effective than samples IP1, IR1 and IR2. 

## 4. Conclusions

The present study investigated the effects of the inlay yarn insertion density levels in different knitted structures used as compression stockings. The compression values and their comfort-related properties were evaluated at the graduated compression points. For healthcare and medical usages, the application of inlay yarn and laid-in stitches is crucial in order to provide the desired pressure in preventive stockings. From the results, it was concluded that the developed samples had better comfort-related properties than the commercial sample taken from the market. The pressure was evaluated theoretically using Laplace’s law and estimated experimentally using a medical stocking tester (MST). The correlation was established with an R value of 0.632.

This study utilized a principal component analysis (PCA) for the simultaneous optimization of properties in compression stockings. The simultaneous optimization process suggested that stockings with inlay yarn insertion in alternate courses will show optimum comfort results. The best sample exhibited a maximum air permeability of 700 mm/s, minimum thermal resistance of 0.013 m^2^K/W and overall moisture management capacity (OMMC) of 0.8, showing far superior results to the control sample collected from the market. In this study, a total of 9 principal components were used. PC1 represented the linear combination that explains the maximum variance (43.6%), followed by PC2, PC3 and PC4 (52.1%). The last five PCs (PC5–PC9) contributed to less than 5% of the variance, while the first four PCs represented more than 95% of the total variance among the data set. As per the Kaiser–Guttman principle, the first three principal components (PC1, PC2, PC3) with eigenvalues greater than one were kept for further analysis. 

Consequently, when designing preventive graduated stocking with class 1 compression, it is necessary to choose the most appropriate inlay yarn insertion density and structure combination. This approach is essential in order to improve the wearable comfort properties and to achieve economic benefits by using the minimum amount of inlay yarn, as it contains elastane and is expensive. In the future, more research is necessary to further improve the comfort and dimensional stability of compression stockings by changing the stitch lengths and linear density levels of the different yarns. 

## Figures and Tables

**Table 1:** Yarn specifications.

Sample	Yarn Parameters	Units	Plaiting Yarn (SCV) Linear Density (Denier)	Inlay Yarn DCV Linear Density (Denier)	Main Yarn/Ground Linear Density (Denier)
Developed samples	Overall Linear density	Denier	92 ± 2	320 ± 2	150 ± 2
	Top Covering linear density	Denier	70 ± 1	20 ± 1	-
	Bottom covering linear density	Denier	-	20	-
	Elastane (Lycra) linear density	Denier	20 ± 1	160 ± 2	150 ± 2
	Type	-	Multifilament	Multifilament	Multifilament
	Nylon No. of filaments	-	24	20*2	48
	Composition PU/PA	Fraction	0.08/0.92	0.56/0.44	0.0/1.0
	Color	-	Raw white	Raw white	Raw white
	Cross section	-	Round	Round	Round

**Table 2:** Experimental samples and codes.

Sample No.	Sample Code	Sample Name/ID
1	P	Plain
2	R1	Rib 2 × 2
3	R2	Rib 3 × 1
4	IP1	insertion every course Plain
5	IR1	Insertion Rib 2 × 2
6	IR2	Insertion Rib 3 × 1

**Table 3:** Structural representation and systematic diagrams of the developed samples.

Sample Code	Sample Id	Loop Structure	Fabric View		Microscopic View	
			Front	Back	Front	Back
P	Plain	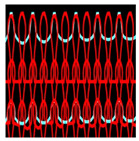	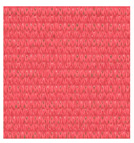	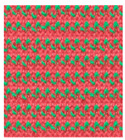	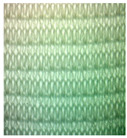	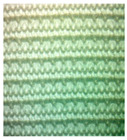
R1	Rib 2 × 2	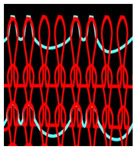	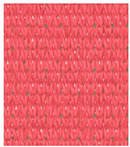	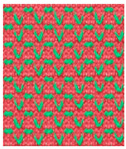	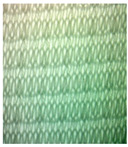	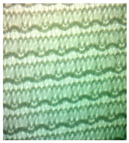
R2	Rib 1 × 3	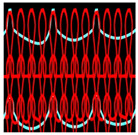	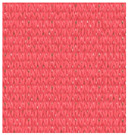	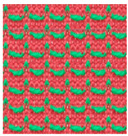	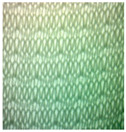	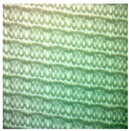
IP1	Insertion Plain 1 × 1	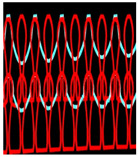	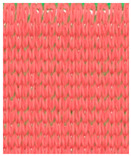	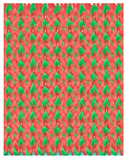	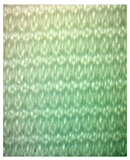	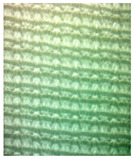
IR1	Insertion Rib 2 × 2	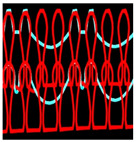	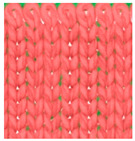	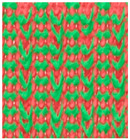	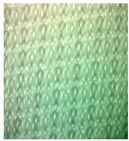	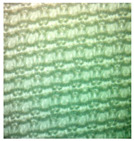
IR2	Insertion Rib 1 × 3	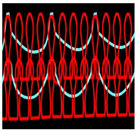	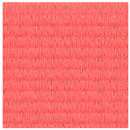	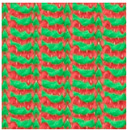	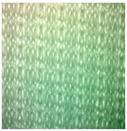	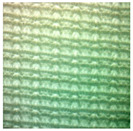

**Table 4:** Gradients of the studied compression stockings [18,27].

Sr. No	Pressure Points	Required Pressure in Percentage
1	B (ankle)	100
2	B1	70 to 100
3	C	50 to 80
5	D, E, F, G	20 to 60

**Table 5:** Details of experiments and mean experimental results.

Experimental Run	Sample	Responses								
	Sample Code	Y1: Compression by MST (mmHg)	Y2: AP (mm/s)	Y3: TR (m^2^K/W)	Y4: OMMC	Y5: Stretch (%)	Y6: Recovery (%)	Y7: Shrinkage (%)	Y8: Thickness (mm)	Y9: Areal Density (g/m^2^)
1	P	19.4	534.06	0.03	0.58	63.73	98.55	−2.5	0.75	221
2	R1	21.1	572.07	0.02	0.59	65.03	98.55	−1.31	0.94	220
3	R2	18.7	689	0.01	0.79	62.5	100	0	0.99	239
4	IP1	24.5	488	0.02	0.64	62.86	94.69	−2.5	0.71	257
5	IR1	20.03	504.03	0.02	0.71	63.42	100	0	0.9	260
6	IR2	22	627.05	0.04	0.77	62.5	96	−6.25	0.87	252

**Table 6:** Average pressure values.

Sample Code	Measuring Points	Compression Values (mmHg)
C	B	20.1
	B1	14.2
	C	13.5
T	B	16.1
	B1	14.9
	C	12.5
P	B	19.4
	B1	14.3
	C	13.2
R1	B	21.1
	B1	15.4
	C	11.1
R2	B	18.7
	B1	12.5
	C	10.7
IP1	B	24.5
	B1	16.8
	C	11.2
IR1	B	20
	B1	17
	C	12
IR2	B	22
	B1	17.3
	C	13.7

Note: The pressure exerted on the leg: 1 mmHg = 133.3 Pa.

**Table 7:** Comparison of results using both methods.

Sample Code	By MST MKV	By Laplace Equation (mmHg)	Difference	Deviation %
P	19.4	19.66	−0.26	−1.34021
R1	21.1	20.95	0.15	0.7109
R2	18.7	17.22	1.48	7.914439
IP	24.5	30.02	−5.52	−22.5306
IR1	20	31.52	−11.52	−57.6
IR2	22	28.08	−6.08	−27.6364

**Table 8:** Signal-to-noise (S/N) ratios of the responses.

	Factor	Responses								
Run	Sample Code	Compression (mmHg)	AP (mm/s)	TR (m^2^K/W)	OMMC	Stretch (%)	Recovery (%)	Shrinkage (%)	Thickness (mm)	Areal Density (g/m^2^)
1	P	25.76	54.55	0.03	−4.79	36.09	39.87	−7.96	2.54	−46.89
2	R1	26.49	55.15	0.02	−4.64	36.26	39.87	−2.33	0.57	−46.85
3	R2	25.44	56.76	0.01	−2.05	35.92	40.00	−1.58	0.12	−47.57
4	IP1	27.78	53.77	0.02	−3.93	35.97	39.53	−7.96	3.01	−48.20
5	IR1	26.03	54.05	0.02	−3.02	36.04	40.00	−1.58	0.92	−48.30
6	IR2	26.85	55.95	0.04	−2.24	35.92	39.65	−15.92	1.24	−48.03

**Table 9:** Normalized S/N ratio.

	Factor	Responses								
Run	Sample Code	Compression (mmHg)	AP (mm/s)	TR (m^2^K/W)	OMMC	Stretch (%)	Recovery (%)	Shrinkage (%)	Thickness (mm)	Areal Density (g/m^2^)
1	P	0.136	0.261	0.546	0.000	0.492	0.732	0.445	0.165	0.027
2	R1	0.447	0.461	0.257	0.055	1.000	0.732	0.052	0.844	0.000
3	R2	0.000	1.000	0.000	1.000	0.000	0.993	0.000	1.000	0.496
4	IP1	1.000	0.000	0.415	0.315	0.145	0.000	0.445	0.000	0.931
5	IR1	0.254	0.094	0.164	0.646	0.369	1.000	0.000	0.724	1.000
6	IR2	0.602	0.727	1.000	0.933	0.001	0.252	1.000	0.611	0.813

**Table 10:** Eigenvalues of the six remaining objectives.

	PC1	PC2	PC3	PC4	PC5	PC6
Eigenvalue	3.9269	2.7173	1.4085	0.5704	0.377	0
Proportion	0.436	0.302	0.156	0.063	0.042	0
Cumulative	0.436	0.738	0.895	0.958	1	1

**Table 11:** Principal component analysis matrix.

Principal Component	Eigen Value	Proportion	Eigen Vector								
First	3.9269	0.436	0.425	0.306	−0.385	−0.635	0.482	0.476	−0.413	0.383	−0.225
Second	2.7173	0.302	0.1	−0.407	−0.079	−0.598	0.5	−0.021	−0.16	−0.274	−0.331
Third	1.4085	0.156	−0.193	0.451	0.472	−0.108	0.164	−0.031	0.412	0.1	−0.563

**Table 12:** Normalized S/N ratios and MRPI values.

	Factor	Responses										
Run	Sample Code	Compression (mmHg)	AP (mm/s)	TR (m^2^K/W)	OMMc	Stretch (%)	Recovery (%)	Shrinkage (%)	Thickness (mm)	Areal Density (g/m^2^)	MRPI Values	Order
1	P	0.136	0.261	0.546	0.000	0.492	0.732	0.445	0.165	0.027	0.386	3
2	R1	0.447	0.461	0.257	0.055	1.000	0.732	0.052	0.844	0.000	1.330	1
3	R2	0.000	1.000	0.000	1.000	0.000	0.993	0.000	1.000	0.496	0.415	2
4	IP1	1.000	0.000	0.415	0.315	0.145	0.000	0.445	0.000	0.931	−0.258	5
5	IR1	0.254	0.094	0.164	0.646	0.369	1.000	0.000	0.724	1.000	0.369	4
6	IR2	0.602	0.727	1.000	0.933	0.001	0.252	1.000	0.611	0.813	−0.741	6

## Data Availability

Not applicable.
